# Supplementary material for: Problems in using p-curve analysis and text-mining to detect rate of p-hacking and evidential value
Source: PeerJ. 2016 Feb 18;4:e1715. doi: 10.7717/peerj.1715 (PMC4768688; doi:10.7717/peerj.1715)
Supplement: Appendix S1 [file peerj-04-1715-s001.pdf]

SUPPLEMENTAL FILES

APPENDIX 1 TO 3

Problems in using p-curve analysis and text-mining to detect rate of p-hacking and evidential value

D. V. M. Bishop[1] & Paul A. Thompson
University of Oxford, UK

Appendix 1

Figure A1: Schematic illustrating simulation of data by the Ghostphack program, with effect size = 1.

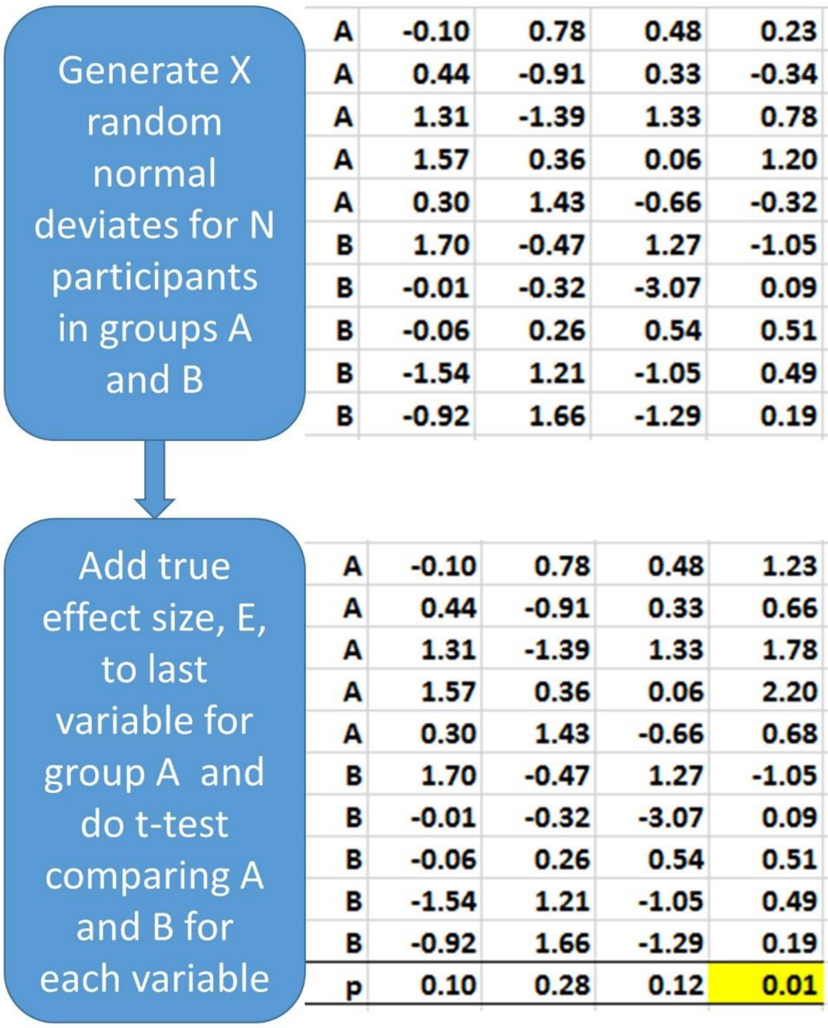

A	-0.10	0.78	0.48	0.23
A	0.44	-0.91	0.33	-0.34
A	1.31	-1.39	1.33	0.78
A	1.57	0.36	0.06	1.20
A	0.30	1.43	-0.66	-0.32
B	1.70	-0.47	1.27	-1.05
B	-0.01	-0.32	-3.07	0.09
B	-0.06	0.26	0.54	0.51
B	-1.54	1.21	-1.05	0.49
B	-0.92	1.66	-1.29	0.19

A	-0.10	0.78	0.48	1.23
A	0.44	-0.91	0.33	0.66
A	1.31	-1.39	1.33	1.78
A	1.57	0.36	0.06	2.20
A	0.30	1.43	-0.66	0.68
B	1.70	-0.47	1.27	-1.05
B	-0.01	-0.32	-3.07	0.09
B	-0.06	0.26	0.54	0.51
B	-1.54	1.21	-1.05	0.49
B	-0.92	1.66	-1.29	0.19
p	0.10	0.28	0.12	0.01

Appendix 2: Plots from Ghostphack to complement Figure 2.

Figure A2: Simulation as for Figure 2, but with no Ghostphacking. Note that amount of covariance between variables has no effect in this situation and so all curves for a given N are superimposed.

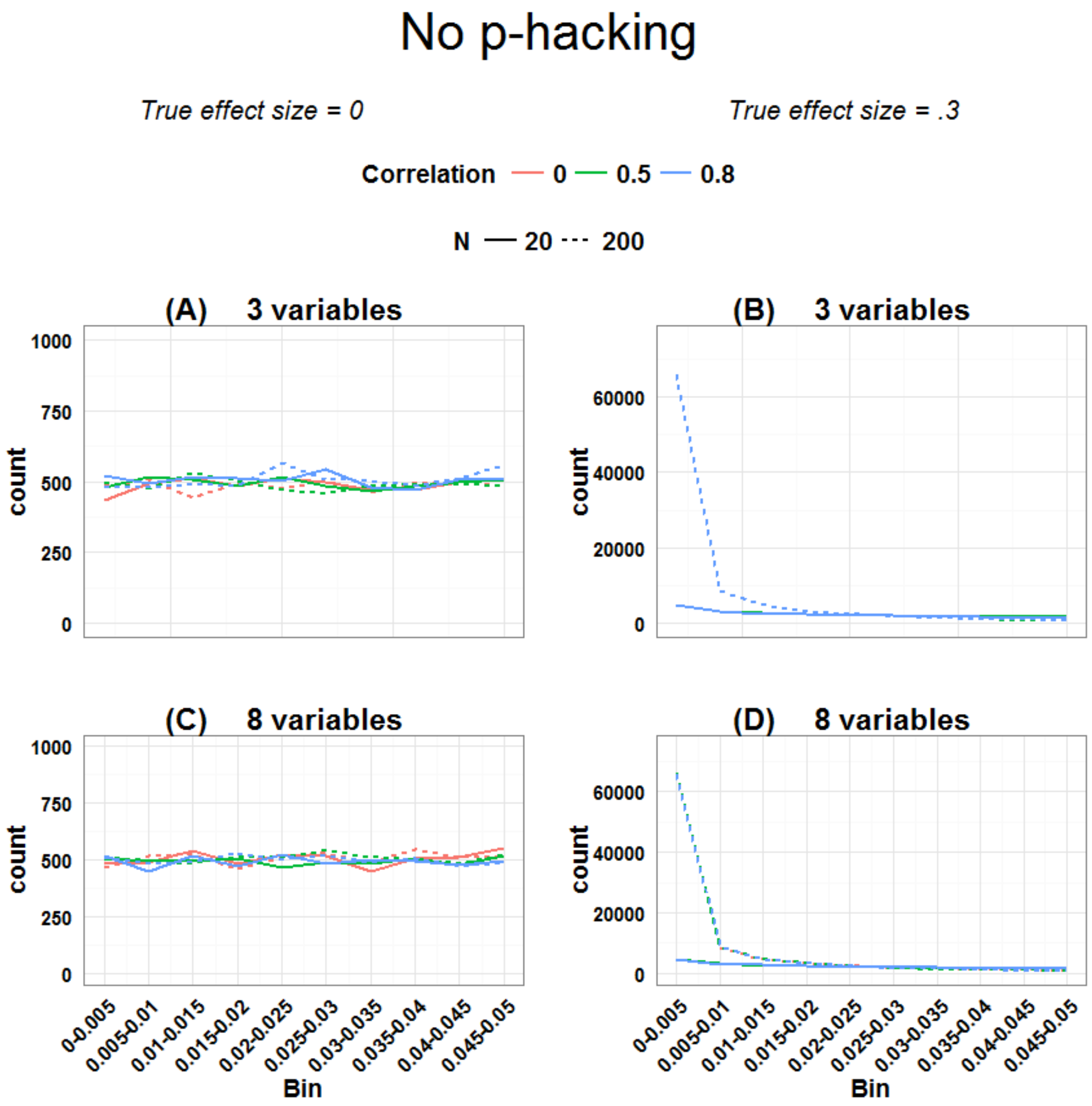

Figure A3: Simulation as for Figure 2, with y-axis as percentage of all p-values, rather than frequency

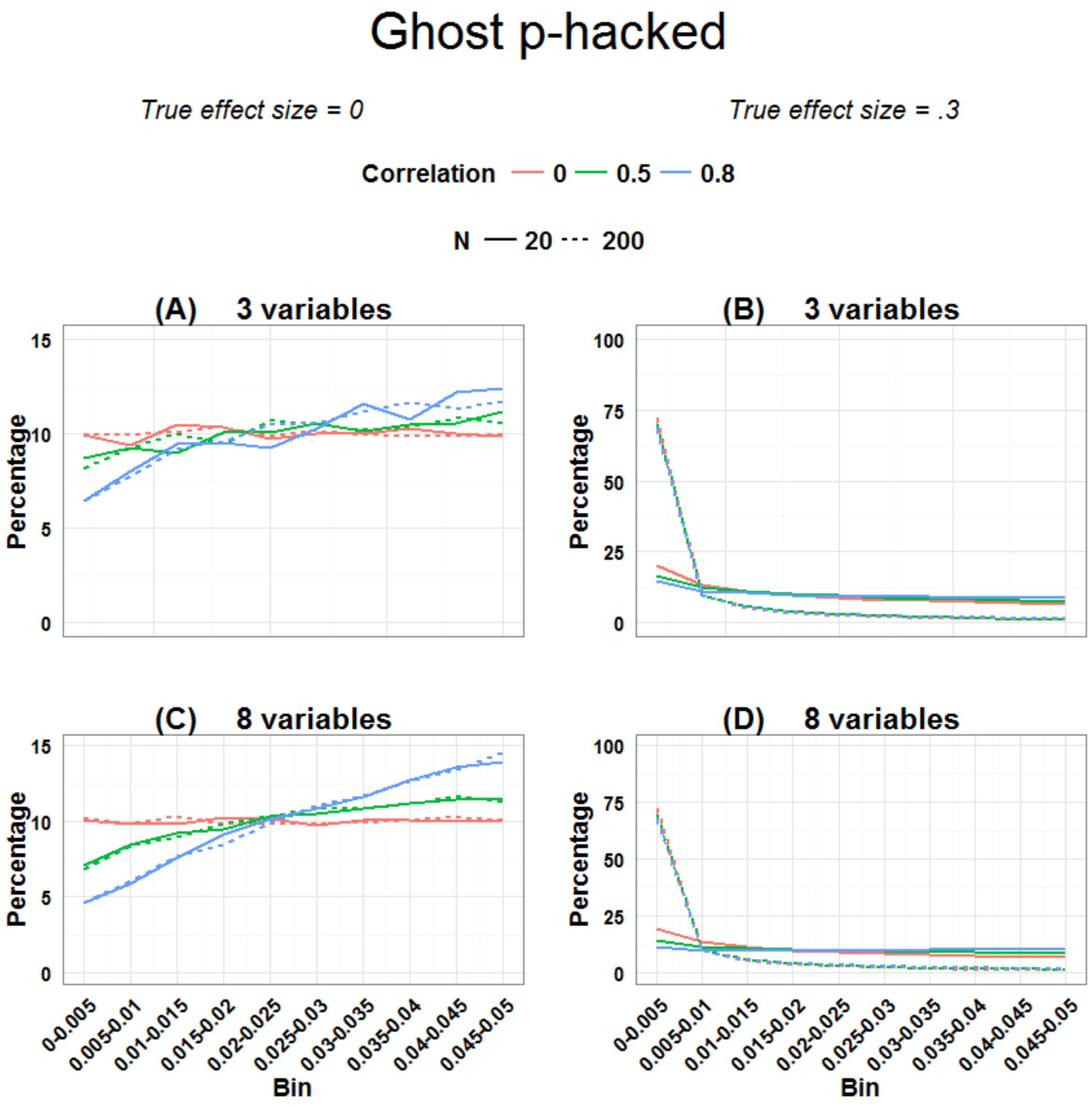

Figure A4: Simulation as for Plot A2, with y-axis as percentage of all p-values, rather than frequency

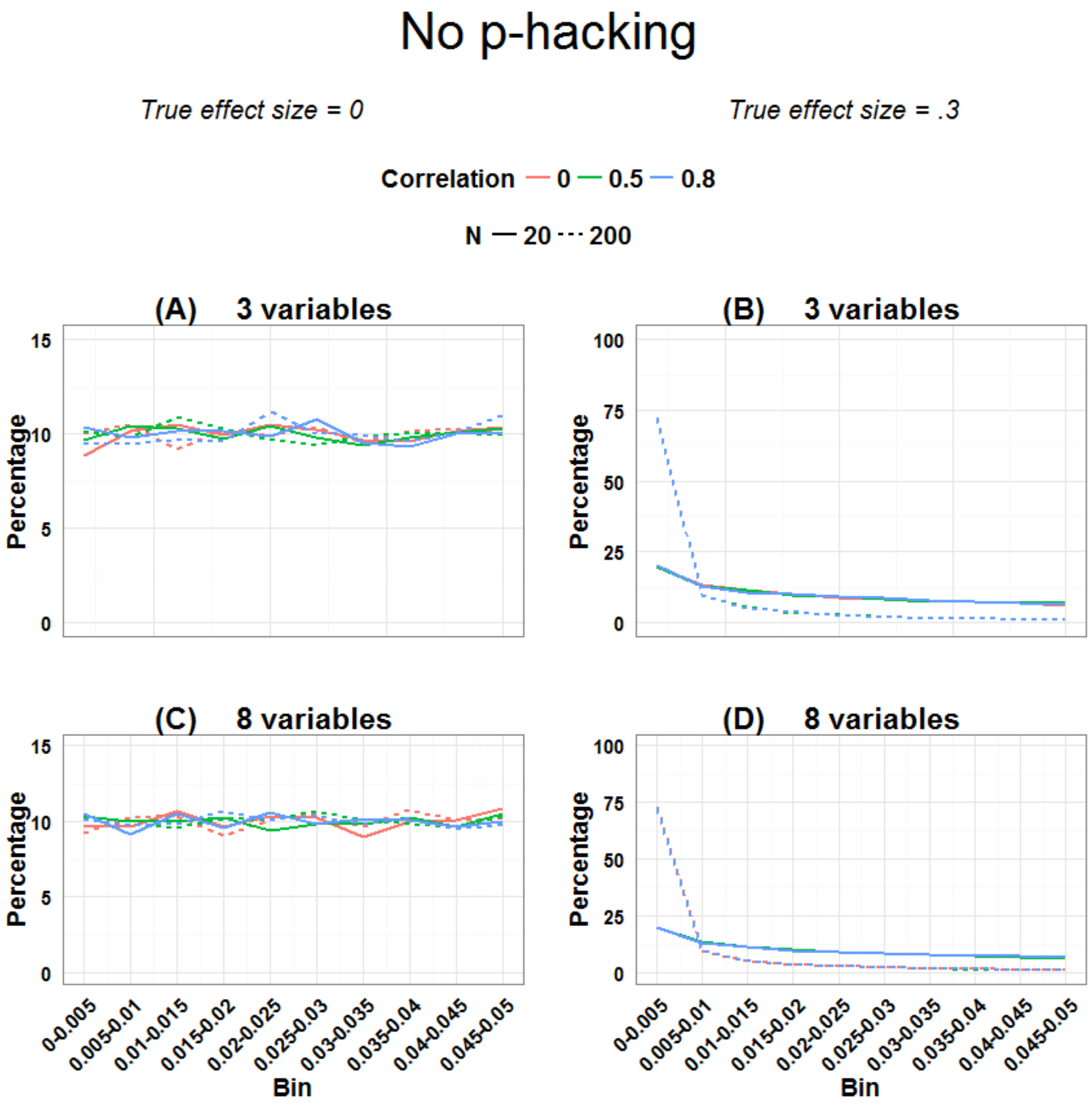

Appendix 3

DOIs of 30 psychology papers included in the Head et al. 2015 analysis that were scrutinised for qualitative analysis of p-values.

Code	first.doi
1	10.1186/1744-859X-12-15
2	10.3758/s13414-010-0033-2
3	10.1186/1744-9081-1-22
4	10.1186/1744-9081-4-36
5	10.1186/1744-9081-3-40
6	10.1186/1744-9081-5-30
7	10.1186/1744-9081-5-16
8	10.1186/1744-9081-6-7
9	10.1186/1744-9081-7-18
10	10.1186/1744-9081-10-10
11	10.1186/2045-5380-2-22
12	10.1111/j.1467-7687.2007.00620.x
13	10.1037/a0016305
14	10.3389/fpsyg.2012.00023
15	10.3389/fpsyg.2012.00533
16	10.3389/fpsyg.2012.00352
17	10.3389/fpsyg.2013.00942
18	10.3389/fpsyg.2013.00015
19	10.3389/fpsyg.2013.00452
20	10.3389/fpsyg.2012.00081
21	10.3389/fpsyg.2014.00276
22	10.3389/fpsyg.2014.00367
23	10.3389/fpsyg.2014.00170
24	10.3389/fpsyg.2014.00430
25	10.3389/fpsyg.2011.00319
26	10.3389/fpsyg.2013.00110
27	10.1016/j.jesp.2013.05.008
28	10.1186/1747-597X-8-20

29 10.1186/1747-597X-9-13

30 10.1186/1747-597X-1-27
